# Impact of Total Laboratory Automation on Turnaround Times for Urine Cultures and Screening Specimens for MRSA, ESBL, and VRE Carriage: Retrospective Comparison With Manual Workflow

**DOI:** 10.3389/fcimb.2020.552122

**Published:** 2020-10-28

**Authors:** Abdessalam Cherkaoui, Gesuele Renzi, Romain Martischang, Stephan Harbarth, Nicolas Vuilleumier, Jacques Schrenzel

**Affiliations:** ^1^Bacteriology Laboratory, Division of Laboratory Medicine, Department of Diagnostics, Geneva University Hospitals, Geneva, Switzerland; ^2^Infection Control Program, Geneva University Hospitals and Faculty of Medicine, Geneva, Switzerland; ^3^Division of Laboratory Medicine, Department of Diagnostics, Geneva University Hospitals, Geneva, Switzerland; ^4^Division of Laboratory Medicine, Department of Medical Specialties, Geneva University Hospitals and Faculty of Medicine, Geneva, Switzerland; ^5^Genomic Research Laboratory, Division of Infectious Diseases, Department of Medical Specialties, Geneva University Hospitals and Faculty of Medicine, Geneva, Switzerland

**Keywords:** turnaround-times, WASPLab, total laboratory automation, reduction of time to report, urine culture, ESBL - extended-spectrum beta-lactamase, MRSA - methicillin-resistant *Staphylococcus aureus*, VRE - vancomycin-resistant enterococcus

## Abstract

Using computerized time-stamps, we compared the turnaround-times (TAT) for urine samples and screening ESwabs of MRSA, VRE, and ESBL carriage in the bacteriology laboratory of Geneva University Hospitals between January and December 2017 (period preceding the implementation of the WASPLab^TM^) with the same specimen types analyzed between January and December 2019 (period after the implementation of the automation). During both 1-year periods, a total of 98'380 specimens were analyzed (48'158 in 2017 vs. 50'222 in 2019). On the WASPLab^TM^, all culture plates were imaged at defined intervals each day of incubation, but the processing of the cultures (i.e., pathogen identification and antimicrobial susceptibility testing) was only performed during day shift hours (~8:00 A.M. to 4:30 P.M.). The median TAT for negative reports decreased by almost half for urine samples from 52.1 (2017) to 28.3 h (2019) (*p* < 0.001), and for MRSA screening specimens from 50.7 to 26.3 h (*p* < 0.001). The difference in median TAT for negative reports was less pronounced for screening of ESBL (50.2 vs. 43.0 h) (*p* < 0.001) and VRE (50.6 vs. 45.7 h) (*p* < 0.001). Despite a trend toward shorter result delivery for positive samples, there was no significant change in the median TAT. These results suggest that TAT for negative samples immediately benefit from automation, whereas TAT for positive samples also depend on the laboratory hours of operation and daily human resource management.

## Introduction

Tremendous resources were allocated during the last two decades to develop faster assays and optimized microbiological processes, with the ultimate goal to expedite answers to the ordering clinicians, as measured by reduced turnaround-times (TAT) a major requisite for timely patient management in case of infection suspicion (Francois et al., 2007; Afshari et al., 2012). Conventionally, the workup of microbial cultures in routine clinical laboratories is tightly related to the laboratory hours of operation and to the daily human resource management, much more than driven by the growth kinetics of the microorganisms. This can be explained by the diversity of the samples that has led to a cascade of complex analytical processes, most of them performed manually. In essence, clinical samples are inoculated onto various agar media according the origin of the specimen. Culture media plates are then transferred manually into conventional incubators and removed the next morning to be inspected by technologists. This way of working, which was the norm until recently, can now be deeply revisited due to the availability of total laboratory automation (TLA) systems for culture-based bacteriology. TLA enables sample streaking and swift transfer into incubators with more stable temperature control. The availability of automated digital pictures of culture plates at defined time-points, as specified by the user, obviates the classical 24 h culture cycle. TLA had therefore an immediate and significant impact by enabling standardized and shorter durations of cultures (Dauwalder et al., 2016; Graham et al., 2016; Burckhardt et al., 2018, 2019; Cherkaoui et al., 2020a). Our laboratory implemented the WASPLab^TM^ system in November 2018. The whole automation process was split into different steps (based on the various specimen types) allowing sequential validation of each process (e.g., defining accurate and reproducible time points for plate imaging and for closing the case), thereby providing documentation for the accreditation, a non-disruptive training of the technologists and a stepwise routine implementation (Cherkaoui et al., 2020b).

The purpose of the current study was to compare the direct impact of implementing the WASPlab^TM^ on the TAT (i.e., the time needed to process the sample from reception in the laboratory to the delivery of the culture results to the prescribing physician) taken to complete the analysis process within the lab for urine samples, as well as for nasal and inguinal/perineal screening-ESwab for methicillin-resistant *Staphylococcus aureus* (MRSA), rectal screening-ESwab for extended-spectrum beta-lactamase-producers (ESBLs) and vancomycin-resistant *Enterococcus* (VRE).

## Materials and Methods

### Setting

The present study was performed in the bacteriology laboratory of Geneva University Hospitals, a Swiss tertiary care center with 1'920 beds. The bacteriology laboratory processes ~180'000 clinical samples annually including 30'000 urines, and 28'000 ESwabs for the screening of MRSA, VRE, and ESBL carriage. Current hours of operation of the bacteriology laboratory span from 07.30 A.M. to 10.00 P.M. (7/7). On the WASPLab^TM^, all culture plates were imaged at defined intervals each day of incubation, but the processing of the cultures [i.e., pathogen identification and antimicrobial susceptibility testing (AST)] is currently only carried out during the day shift (~8:00 A.M. to 4:30 P.M.).

### Study Design

We compared the TAT for urine cultures, and for the screening of MRSA, VRE and ESBLs carriage for the specimens referred to the bacteriology laboratory between January and December 2017 and analyzed using the WASP coupled to conventional incubation and manual diagnostic (period preceding the implementation of the WASPLab^TM^) with the same specimens types that were analyzed using the WASPLab^TM^ between January and December 2019. We began using automation on such specimens in November 2018. However, in order to minimize the effects of the run-in period for using the WASPLab^TM^, we have chosen to start our observational study on January 2019. To avoid results variability for urine cultures between 2017 and 2019, we restricted the comparison of TAT for negative specimens, the positive specimens with mixed flora, and monomicrobial cultures with the frequent uro-pathogens (*Escherichia coli*, and *Enterococcus faecalis*). All bacterial culture results and time-stamps were downloaded from our in-house laboratory information system (Unilab2, version 2.38.1). Table 1 depicts the number of samples included by specimen type, the culture procedures, and the analysis parameters defined on the WASPLab^TM^ based on our previous studies (Cherkaoui et al., 2019a,b). During the two compared periods, the identification of the bacterial strains was performed by matrix-assisted desorption ionization time-of-flight mass spectrometry (MALDI-TOF/MS, Bruker Daltonics, Bremen, Germany) according to the manufacturer's instructions. The antimicrobial susceptibility testing was performed by disc diffusion and VITEK2 (BioMérieux) according to the EUCAST guidelines. All the negative results were validated and transmitted to the clinicians by the technologists (i.e., not auto-released by the WASPLab^TM^). The positive results were not reported based on specific color on chromogenic media, thus confirmatory testing was systematically performed. The presence of ESBL was confirmed by double-disc synergy tests (DDST20 and DDST30). Confirmation of MRSA and MSSA strains was performed by a previously published qPCR assay targeting *fem*A and *mec*A (Francois et al., 2003). The presence of VRE was confirmed by defining the minimum inhibitory concentration (MIC) for vancomycin and teicoplanin.

**Table 1:** Workup of bacterial culture, samples included in this study, and analysis parameters on the WASPLab^TM^ based on previous studies.

**Clinical sample type**	**Solid culture media type**	**Number of samples included in this study**		**% of positive samples**		**WASP coupled to conventional incubation and manual diagnostic**	**Time points for digital images acquisition on WASPLab^TM^**			
		**2017**	**2019**	**2017**	**2019**	**Incubation period (h)**	**Picture at T0**	**First time point, (h)**	**Second time point, (h)**	**Final time point, (h)**
Urine specimens	CHROMID® CPS® Elite (BioMérieux)	19,929	18,217	50.6% (10,078/19,929)	51.3% (9,338/18,217)	18–24 and 48	Yes	18	No	24
Nasal and inguinal/perineal screening-Eswab for MRSA	CHROMID® MRSA (BioMérieux)	18,459	15,901	4.1% (751/18,459)	5.2% (826/15,901)	18–24 and 48	Yes	No	No	18
Rectal screening-Eswab for ESBL	CHROMID® ESBL (BioMérieux)	7,797	8,640	27.5% (2,140/7,797)	25.4% (2,196/8,640)	18–24 and 48	Yes	No	No	16
Rectal screening-Eswab for VRE	CHROMID® VRE (BioMérieux)	1,973	7,464	2.3% (45/1,973)	0.8% (58/7,464)	18–24 and 48	Yes	18	24	30

January to December 2017: The analyses were performed using the WASP coupled to conventional incubation and manual diagnostic (period preceding the implementation of the WASPLab^TM^).

*January to December 2019: period after the implementation of the WASPLab^TM^*.

### Statistical Analyses

Differences in TAT were reported as the median to account for skewed distribution, and tested using Wilcoxon test. All analyses were performed using R.3.6, and RStudio software [RStudio Team (2015)].

## Results

### Urine Cultures

**Figure 1:**
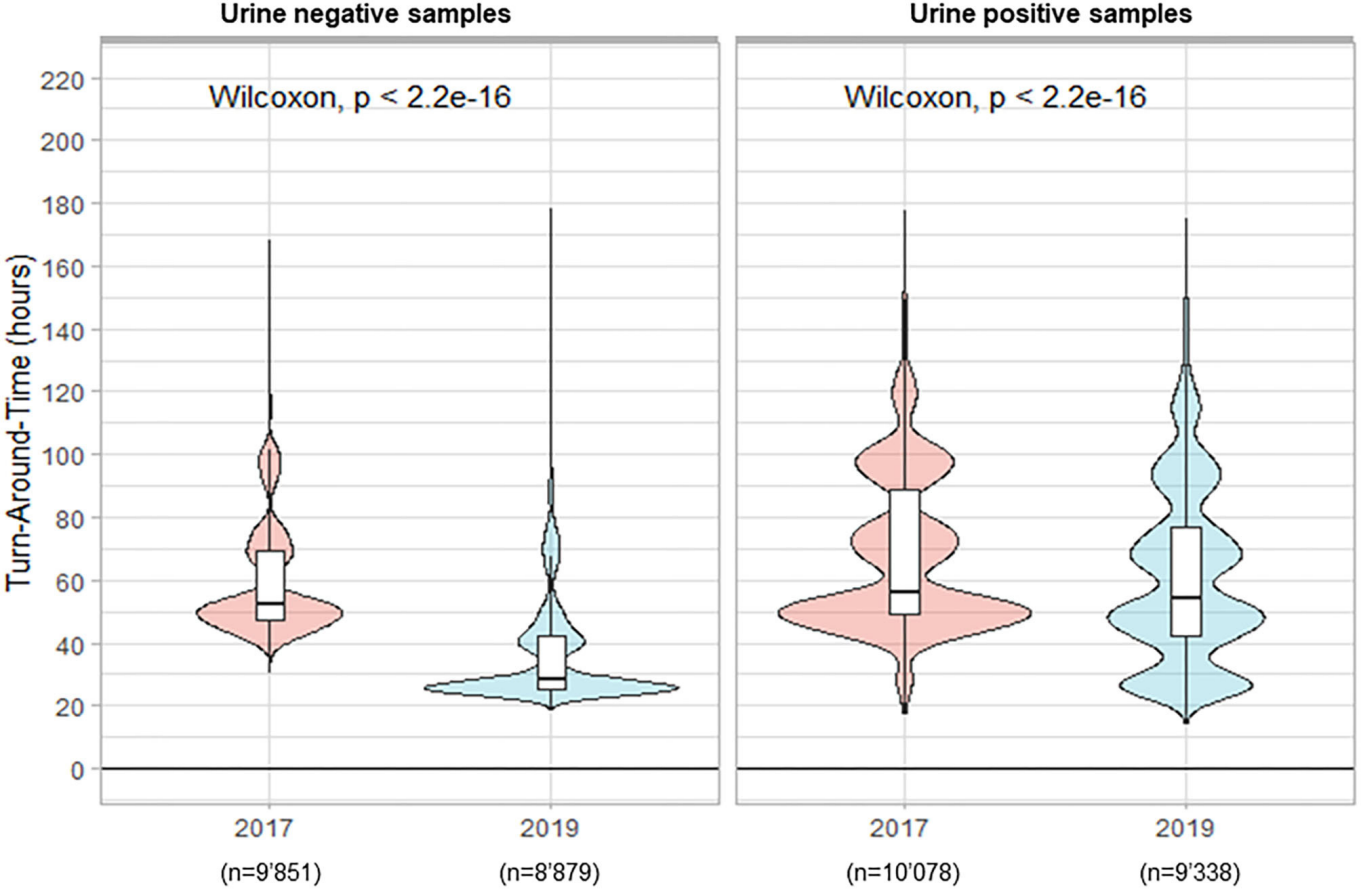
Turn-Around Times (from reception of samples to delivery of the culture results) for urine analysis.

For the positive samples, only cultures positive with mixed flora, *Escherichia coli*, or *Enterococcus faecalis* were included in the analysis. In total, 19'929 urine samples were cultivated in 2017 vs. 18'217 samples in 2019. The percentage of positive samples remained stable around 51%. During the period preceding TLA, the median TAT for negative results was 52.1 h. The implementation of the WASPLab^TM^ significantly reduced the TAT to 28.3 h (*p* < 0.001) in 2019 (Figure 1). Importantly, we did not see any significant decrease in the TAT for positive samples (56.2 vs. 54.0 h; *p* < 0.001). The culture workup (i.e., uropathogen identification and AST) was still performed on the day shifts and only during weekdays except for urine samples from infants and pregnant women (about 2–3% of the samples).

### Screening Specimens for MRSA Carriage

**Figure 2:**
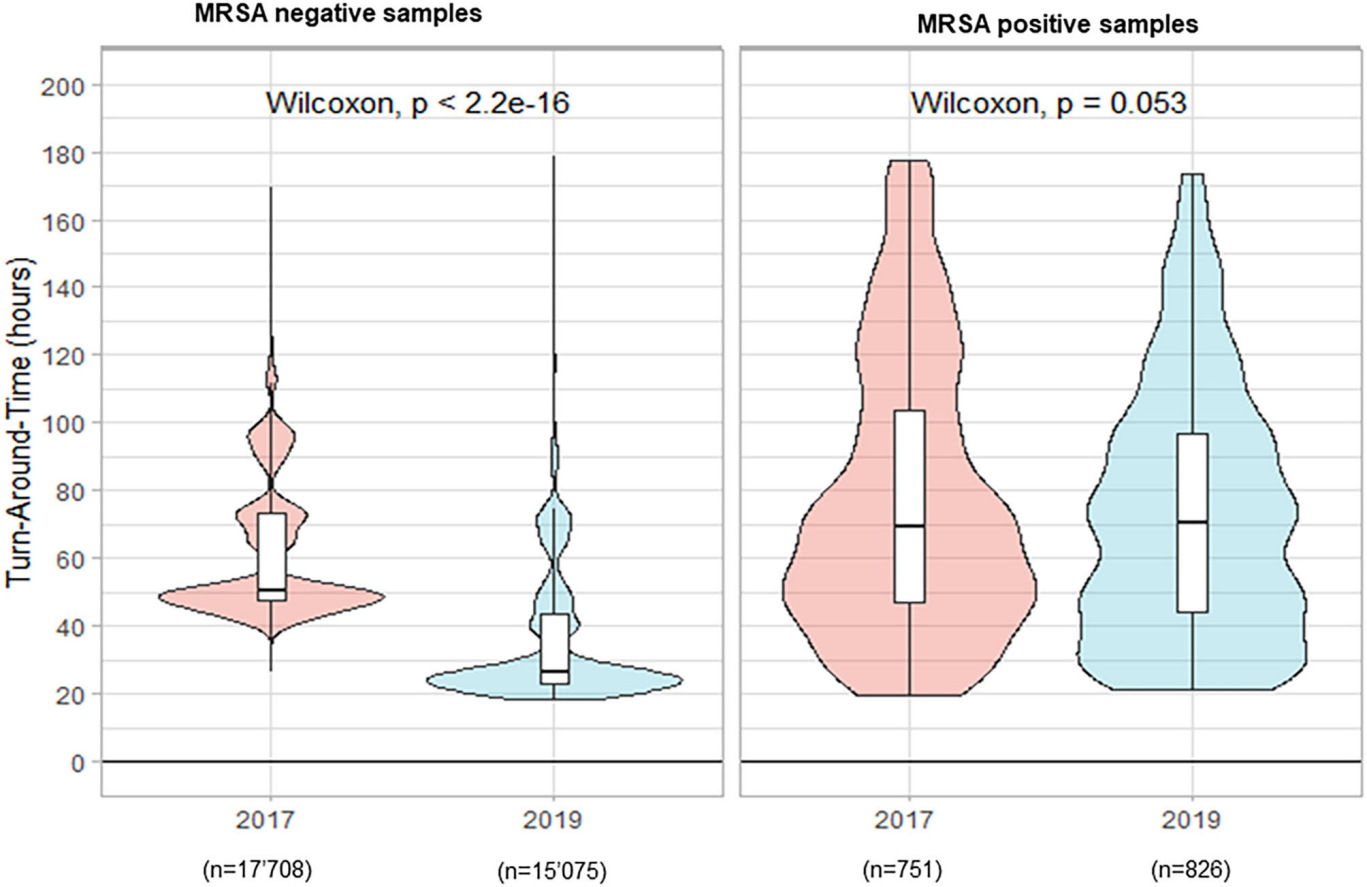
Turn-Around Times (from reception of samples to delivery of the culture results) for nasal and inguinal/perineal screening-ESwab for methicillin-resistant *Staphylococcus aureus* (MRSA) carriage by culture.

In total 18'459 nasal and inguinal/perineal samples were analyzed in 2017 to detect MRSA carriage vs. 15'901 samples in 2019. The percentage of positive samples reached 4.1% in 2017 and showed a slight increase in 2019, with 5.2% positivity. During the period preceding TLA, the median TAT for negative results was 50.7 h. The implementation of the WASPLab^TM^ with the incubation times defined and previously validated (Cherkaoui et al., 2019a,b) enabled a significant reduction of the median TAT to 26.3 h (*p* < 0.001) (Figure 2). However, we did not see any decrease in the median TAT for positive samples (69.2 h in 2017 vs. 70.2 h in 2019, *p* = 0.053).

### Rectal ESwabs for Detection of ESBL Carriage

**Figure 3:**
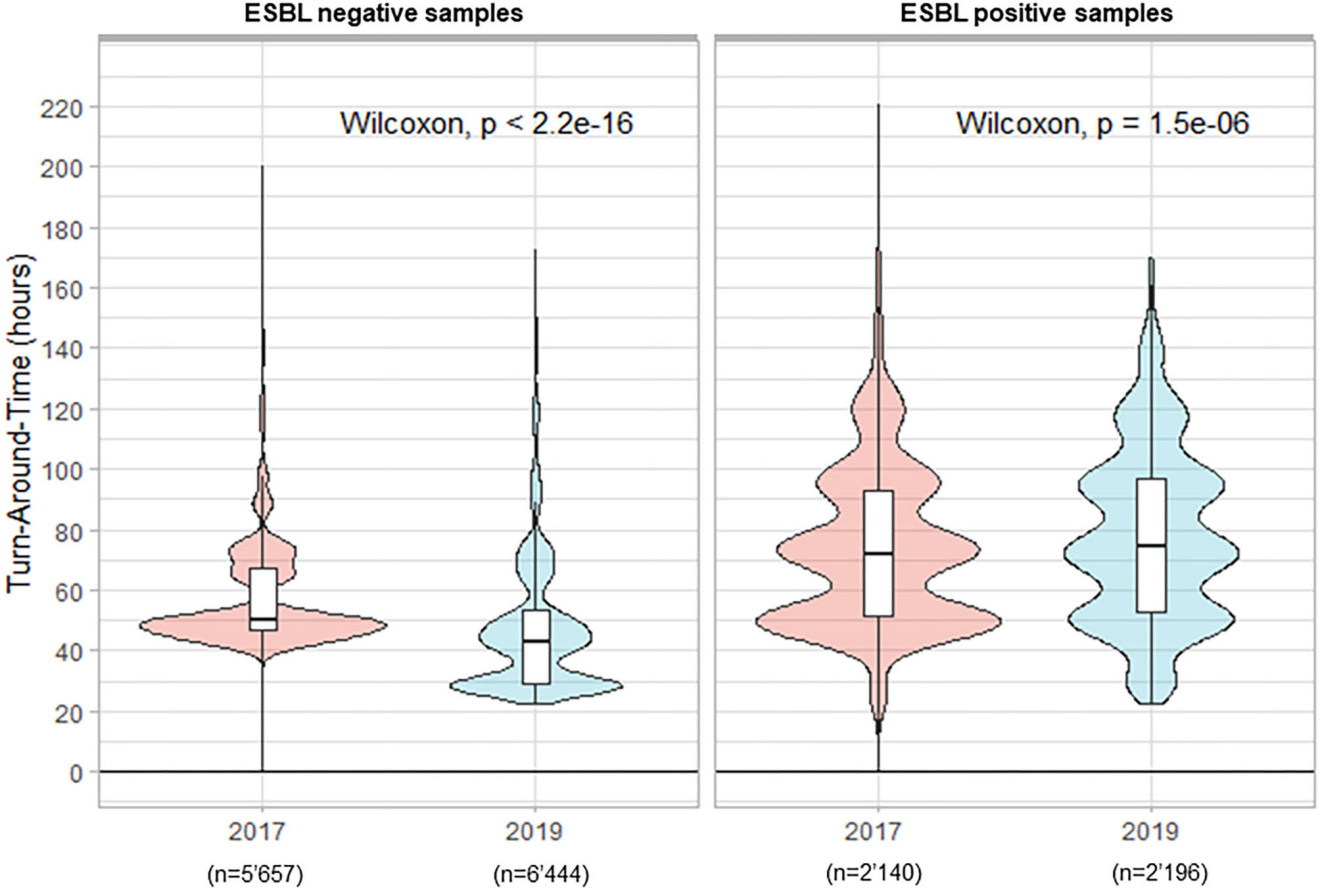
Turn-Around Times (from reception of samples to delivery of the culture results) for rectal screening-ESwab for extended-spectrum beta-lactamases (ESBLs) carriage by culture.

In total 7'797 rectal ESwab were analyzed in 2017 for the screening of ESBL carriage vs. 8'640 samples in 2019. The percentage of positive samples was 27.5 and 25.4%, in 2017 and 2019, respectively. During the period preceding TLA, the median TAT for negative results was 50.2 h. The implementation of TLA with its previously defined timing parameters led to a statistically significantly reduced median TAT of 43.0 h (*p* < 0.001) in 2019 (Figure 3). Paradoxically, we witnessed an increased median TAT for positive samples (from 72.2 h in 2017 to 74.4 h in 2019; *p* < 0.001).

### Detection of VRE From Rectal ESwab Samples

**Figure 4:**
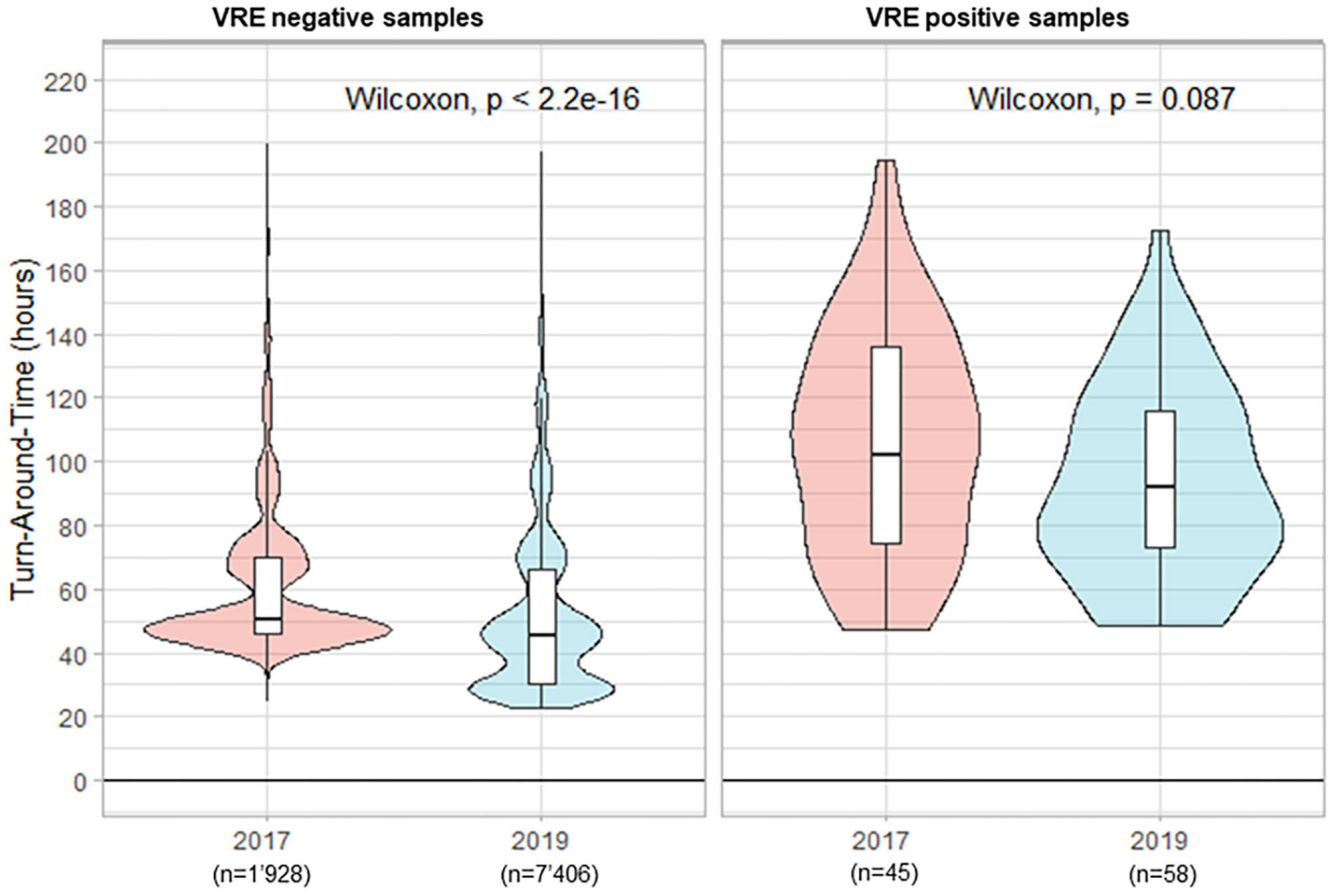
Turn-Around Times (from reception of samples to delivery of the culture results) for rectal screening-ESwab for vancomycin-resistant *Enterococcus* (VRE) carriage by culture.

In total 1'973 rectal ESwabs were processed in 2017 for the screening of VRE carriage vs. 7'464 samples in 2019. The percentage of positive samples decreased from 2.3 to 0.8% in 2017 and 2019, respectively. The important increase in samples referred to the laboratory in 2019 was related to the implementation of a systematic screening in 2019, whereas in 2017 the search for VRE was performed only for suspected cases. This change toward a systematic screening was prompted by a nosocomial VRE outbreak in Switzerland (Moulin et al., 2018; Buetti et al., 2019). During the period preceding the automation, the median TAT for negative results was 50.6 h (Figure 4). The implementation of the WASPLab^TM^ reduced the median TAT slightly to 45.7 (*p* < 0.001). Additionally, we witnessed a reduction of the median TAT for positive samples (from 102.0 to 92.2 h, in 2017 and 2019, respectively; *p* = 0.087), because these samples were also processed during the weekends.

## Discussion

The main objective of a clinical bacteriology laboratory is to provide prompt and accurate results to clinicians and infection control specialists in order to timely target antimicrobial therapy and infection control precautions. In principle, many factors affect the speed of such culture-based results: (i) the microbial growth kinetics, (ii) the specimens' workflow in the laboratory, (iii) the laboratory hours of operation, and (iv) the daily human resource management. The advent of TLA in clinical bacteriology has brought the potential for reduced specimen processing times, more standardized culture-based testing, and decreased TAT. All of that was enabled by the TLA workflow which begins at the inoculation step to permit microorganisms to grow rapidly after inoculation because plates are automatically transferred within a couple of minutes to incubators whose temperature can be kept stable. Importantly, the three steps in the TLA workflow “screening, reading and picking” allow for more flexibility leading to a significant impact on the TAT. As negative results can be determined at the screening phase, without the need for the reading phase, this easily explains why such samples can immediately benefit from reduced TAT. The picking phase and downstream operations for positive samples will therefore rely on the lab workflow organization for improving their TAT.

In our conventional manual workflow, urine cultures and samples for MRSA, ESBL and VRE carriage screening were inoculated 6 days a week (Monday to Saturday), but the reading of culture plates was performed only on weekdays. In addition, all plates were read on two consecutive days, except if the specimen was already reported positive on day 1. This stems from the fact that the inoculation times for individual plates are not recorded, when performing routine manual operations. Additionally, if day 1 and/or day 2 happened to fall on a weekend or a public holiday, the reading of the media plates was deferred to the next working day. With the implementation of the WASPLab^TM^, the incubation times for MRSA, VRE, ESBL, and urines culture media plates could be significantly shortened as evidenced by our previous validation studies (Cherkaoui et al., 2019a,b). Yet, it remained necessary to assess the impact of the WASPLab^TM^ implementation on TAT for urine cultures, and for the screening of MRSA, VRE, and ESBL by culture. Using computerized time-stamps, the duration required from sample reception to results release to the ordering provider was retrospectively assessed for each of the specimens included prior to and after implementation of the WASPLab^TM^. Rates for positive samples remained stable between the pre- and post-TLA study periods, except for VRE because of a Swiss outbreak in 2019 that prompted to add systematic VRE screening in patients at risk in our institution. Importantly, the laboratory hours of operation and the daily human resource management remained unchanged during the compared periods. In particular, culture media plates were read only during the day shift. The median TAT for negative reports went down for MRSA from 50.7 to 26.3 h. Similar results have been previously reported for the detection of MRSA in nasal swabs by Burckhardt et al. (2018), benefiting also from automation. The authors compared the times to report (TTR) for 16'111 nasal swabs (*n* = 7'620 during the classic workflow period and *n* = 8'491 during the TLA; BD Kiestra workflow period). The median TTR for negative reports was reduced by 50%. In contrast with this study, we have not seen any decrease in the median TAT for positive samples, which can be explained by the fact that we did not change neither the daily human resource management in 2019 (i.e., the period after the implementation of the lab automation) nor the laboratory operating hours, as would automation permit.

The culture workup (i.e., MRSA identification by qPCR and AST) was still performed on the day shift and only on weekdays. Additionally, we did not find any significant difference in TAT when we excluded samples referred to the laboratory during weekends. The same trend was observed for urine cultures. Namely, the median TAT for negative reports was reduced by 50% after the implementation of the WASPLab^TM^. These results are similar to findings of previous studies (Yarbrough et al., 2018; Bailey and Burnham, 2019). The difference in the median TAT for negative reports was less pronounced for VRE and ESBL (Table 2). Taken together, this decrease in TAT is directly related to the implementation of the WASPLab^TM^ and to the possible shorter incubation times of the chromogenic agar plates. The use of artificial intelligence (AI), to identify picture plates without bacterial growth could further improve the TAT by providing an automated release of the results, pending an adequate validation of this AI tool.

**Table 2:** Turnaround-times (from reception of samples to delivery of the culture results).

	**Year**	**Urine cultures (h)**		**Screening for MRSA carriage (h)**		**Screening for ESBL carriage (h)**		**Screening for VRE carriage (h)**	
Negative samples	2017	52.1	*P* < 0.001	50.7	*P* < 0.001	50.2	*P* < 0.001	50.6	*P* < 0.001
	2019	28.3		26.3		43.0		45.7	
Positive samples	2017	56.2	*P* < 0.001	69.2	*P* = 0.053	72.0	*P* < 0.001	102.0	*P* = 0.087
	2019	54.0		70.2		74.4		92.2	

Despite a downward trend, there were no significant changes in the median TAT for positive samples (i.e., organism identification and susceptibility results). The slight increase of TAT for ESBL positive samples (72.0 vs. 74.4, in 2017 and 2019, respectively), is probably explained by the introduction in 2018 of a systematic search for ESBLs and carbapenemases for non-fermenting Gram-negative bacilli. Thus, additional time was required for such additional confirmatory tests.

Shortening the TAT could positively improve the patient's outcome and the infection control measures. By providing accurate and earlier results to the physicians, one could contribute to optimize therapeutic decisions. However, the real impact of shorter TAT on medical decisions is strongly linked to the reactiveness of the medical teams when the results are delivered on the laboratory information system. Further studies focusing on the overall effectiveness of earlier reporting of culture results on the patient's outcome are therefore warranted, as well as on the potential improvements on infection control. The implementation of automated image analysis, provided cautious validation, could permit the distribution of alerts (e.g., by SMS) in quasi real-time to the infection control team, and enable to remove unnecessary temporary measures.

## Conclusions

In conclusion, we established here that TLA *per se* contributes to significantly reduce TAT for negative samples of urine cultures and for screening ESwabs of MRSA, VRE, and ESBL carriage. However, TAT for positive samples depends largely on the laboratory organization (hours of operation and daily human resource management). That same analysis has been echoed by a recent report (Dauwalder et al., 2019). TLA may enable improved infection control, by the earlier release of negative screening samples. A more comprehensive lab organization is mandatory to deliver also shorter TAT for positive samples and for guiding better and earlier targeted treatments.

## Data Availability Statement

The raw data supporting the conclusions of this article will be made available by the authors, without undue reservation.

## Ethics Statement

The studies involving human participants were reviewed and approved by the ethics committee Commission cantonale d'éthique de la recherche (CCER) https://www.hug-ge.ch/ ethique. The ethics committee waived the requirement of written informed consent for participation.

## Author Contributions

AC designed the study, analyzed the data, and wrote the manuscript. GR participated in data analysis. RM performed statistical analyses. SH and NV reviewed the manuscript. JS revised the manuscript. All authors contributed to the article and approved the submitted version.

## Conflict of Interest

JS received restricted research grants and participated to advisory boards from BioMérieux and Debiopharm. NV received restricted research grants from Roche. SH participated in an advisory board of DNAe. The remaining authors declare that the research was conducted in the absence of any commercial or financial relationships that could be construed as a potential conflict of interest.
